# Stereotactic Radiosurgery with Neoadjuvant Embolization of Larger Arteriovenous Malformations: An Institutional Experience

**DOI:** 10.1155/2014/306518

**Published:** 2014-01-22

**Authors:** Richard Dalyai, Thana Theofanis, Robert M. Starke, Nohra Chalouhi, George Ghobrial, Pascal Jabbour, Aaron S. Dumont, L. Fernando Gonzalez, David S. Gordon, Robert H. Rosenwasser, Stavropoula I. Tjoumakaris

**Affiliations:** ^1^Department of Neurosurgery, Thomas Jefferson University and Jefferson Hospital for Neuroscience, 901 Walnut Street, 3rd Floor, Philadelphia, PA 19107, USA; ^2^Department of Neurosurgery, University of Virginia, Charlottesville, VA, USA

## Abstract

*Objective.* This study investigates the safety and efficacy of a multimodality approach combining staged endovascular embolizations with subsequent SRS for the management of larger AVMs. *Methods.* Ninety-five patients with larger AVMs were treated with staged endovascular embolization followed by SRS between 1996 and 2011. *Results.* The median volume of AVM in this series was 28 cm^3^ and 47 patients (48%) were Spetzler-Martin grade IV or V. Twenty-seven patients initially presented with hemorrhage. Sixty-one patients underwent multiple embolizations while a single SRS session was performed in 64 patients. The median follow-up after SRS session was 32 months (range 9–136 months). Overall procedural complications occurred in 14 patients. There were 13 minor neurologic complications and 1 major complication (due to embolization) while four patients had posttreatment hemorrhage. Thirty-eight patients (40%) were cured radiographically. The postradiosurgery actuarial rate of obliteration was 45% at 5 years, 56% at 7 years, and 63% at 10 years. In multivariate analysis, larger AVM size, deep venous drainage, and the increasing number of embolization/SRS sessions were negative predictors of obliteration. The number of embolizations correlated positively with the number of stereotactic radiosurgeries (*P* < 0.005). *Conclusions.* Multimodality endovascular and radiosurgical approach is an efficacious treatment strategy for large AVM.

## 1. Introduction

Cerebral arteriovenous malformations (AVM) are rare but potentially devastating vascular lesions that often affect young adults. Intracranial hemorrhage occurs at an average annual rate of 2 to 4% [1]. Surgical excision is the mainstay of treatment for Spetzler-Martin (SM) grade I and II AVM. Because of high procedural morbidity rates, surgery is avoided in larger and higher grade AVM, and alternative therapies are often considered for these lesions.

Radiosurgery, initially conceived by Leksell [2] in 1968, is a well-established treatment alternative to surgical resection for intracranial AVM. However, stand-alone stereotactic radiosurgery (SRS) may have limited utility for larger AVM [3]. For these larger lesions, we have explored a multimodality therapy of initial endovascular embolization for AVM volume reduction followed by SRS to treat the remaining nidus. By reducing the size of the nidus targeted by SRS, endovascular embolization is thought to improve AVM obliteration rates while also minimizing SRS-related complications. Previous series have reported conflicting results regarding the efficacy of preradiosurgical AVM embolization [4]. We report our 15-year experience in managing these challenging lesions with a combined embolization and SRS approach.

## 2. Methods

### 2.1. Patient Selection

**Figure 1:**
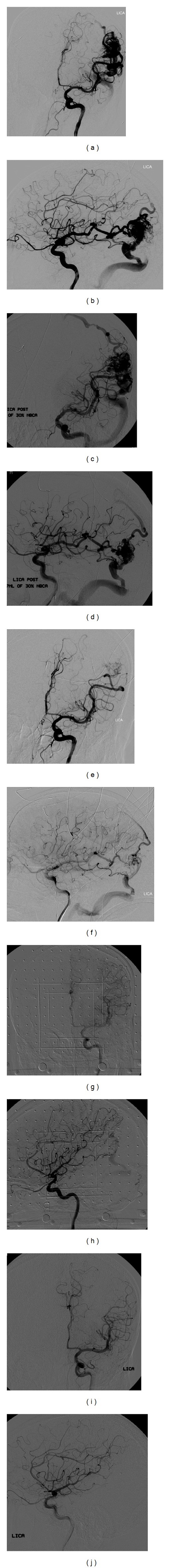
57 yo M presenting with incapacitating migraines found in frontal and lateral views of digital subtraction angiogram, left carotid injection, to have Spetzler-Martin grade III left parietal AVM (a-b). Frontal and lateral views of digital subtraction angiogram showing reduction of AVM nidus after 1st endovascular embolization with NBCA (c-d). Frontal and lateral views of digital subtraction angiogram following third NBCA embolization session with remaining small nidus and fistula (e-f). Frontal and lateral views of digital subtraction angiogram showing small remaining nidus and fistula prior to Gamma Knife SRS planning (g-h). Frontal and lateral views of digital subtraction angiogram showing complete obliteration of AVM 2 years after SRS session (i-j).

An Institutional Review Board approval was obtained prior to data collection. From 1996 to 2011, a total of 775 patients were treated for cerebral AVM in our institution. Of these, 95 patients were selected based on their clinical presentation, SM grade, and angioarchitecture to undergo staged embolization and SRS as seen in Figure 1. We were more likely to aggressively utilize radiosurgery with adjuvant embolization than any treatment at all in those patients with hemorrhagic presentation, SM grade II–IV, and those with arterial pedicles amenable to embolization. All patients had larger AVMs with a maximal diameter greater than 3 cm.

Medical charts, operative reports, SRS records, and pre- and posttreatment imaging results including MR and digital subtraction angiography (DSA) were carefully reviewed to determine patient demographics, AVM characteristics, procedural complications, posttreatment cerebral hemorrhage, radiological AVM obliteration, and neurological outcome.

### 2.2. Embolization Protocol

Embolization was performed with liquid embolic agents NBCA (Codman & Shurtleff, Inc., Raynham, Massachusetts, USA) and, later, Onyx 18 or 34 (eV3, Irvine, CA). Embolization sessions were performed at 6-week intervals, as necessary, until the AVM nidus had been reduced by a goal of 33% (usually to a volume of less than 10 cc). Target selection for AVMs was dependent on two issues, the size of feeding arterial pedicles and the location of arterial feeders on the AVM nidus. Targets were often selected that would treat and obliterate deeper portions of the nidus and those with large volume. Our strategy with embolization was to achieve volume reduction and not flow reduction and ideally to target fixed portions of a nidus that would allow for a discrete nidus when targeting with SRS. Postembolization AVM size was calculated during SRS planning.

### 2.3. Stereotactic Radiosurgery Protocol

A stereotactic head frame was placed for all patients using local anesthesia supplemented by intravenous sedation. Next, biplane digital subtraction stereotactic angiography was performed followed by MR imaging (Figures 1(g) and 1(h)). Volumetric 3D dose planning was performed using Leksell GammaPlan software. For patients with larger lesions and prospectively volume-staged SRS plans, the lesion was divided into approximately equal volumes using anatomic landmarks. SRS treatment plans consisted of the margin SRS dose including the entire AVM nidus volume. SRS was performed with Leksell Gamma Knife units (Elekta AB).

The median initial AVM volume was 28 cm^3^ (largest being 112 cm^3^). The mean Flickinger Pollock grade was 3.65 (range 1.1 to 11.8).

The postembolization mean maximal diameter was 2.2 cm (range 0.3–6 cm). The median total target volume was 4.24 cm^3^ (range 0.26–9.1 cc^3^) on the first SRS treatment and 5.09 cm^3^ on successive SRS treatments. The median margin dose was 21 Gy.

### 2.4. Follow-Up Evaluation

Radiological success was defined as complete AVM obliteration on DSA (total disappearance of the nidus and early draining veins) or, alternatively, on MR angiography (total disappearance of the nidus and flow-voids) for patients with poor overall medical condition or for those refusing follow-up DSA. The median follow-up after SRS session was 32 months (range 9–136 months).

### 2.5. Statistical Analysis

Data are presented as median and range for continuous variables and as frequency for categorical variables. The Flickinger Pollock scale was tested as a continuous variable and as an ordinal variable by quartile. The Radiosurgery AVM Score was tested as previously described [5]. Kaplan Meier analysis was carried out to determine actuarial rate of obliteration. Univariate survival analysis was carried out using the logrank test to test covariates predictive of treatment obliteration. Factors predictive in univariate analysis (*P* < 0.20) were entered into a multivariate Cox proportional hazards model. Interaction and confounding was assessed through stratification and relevant expansion covariates. *P* values of ≤0.05 were considered statistically significant. Statistical analysis was carried out with Stata 10.0 (College Station, TX).

## 3. Results

Ninety-five patients with large and complex AVM underwent preoperative embolization followed by SRS. Patient characteristics are summarized in Table 1. Of the 95 patients, 41 (43%) were women and 54 (57%) were men. The median age was 39 years (range 9–73 years). Thirty-six percent of patients were active smokers and 23% were on oral antihypertensive medication at the time of diagnosis.

AVMs were predominantly located in the left hemisphere (57%). The parietal lobe was the most common location of AVM (27%). The median maximum diameter of AVMs was 4.3 cm. Sixteen (17%) AVM had a maximum diameter greater than 6 cm. The median pretreatment volume was 28 cm^3^. Mean SM grade was 3.6 and almost 50% (47/95) of all lesions were SM grade IV or V. Seventy-one (75%) AVMs were located in eloquent areas and 51 had deep venous drainage (54%). Twenty (29%) patients initially presented with cerebral hemorrhage, and 37 (39%) presented with seizures. The remaining patients were initially diagnosed incidentally or as part of the imaging workup for headaches. In our patient population there were 5 patients with intranidal aneurysms (5%), 1 patient with a high flow fistula (1%), and 9 patients with proximal flow-related aneurysms treated (10%). The mean Flickinger Pollock scale was 3.65 (standard deviation 1.95). The mean Radiosurgery AVM Score was 2.4.

Thirty-four (36%) patients underwent a single embolization, 25 (26%) patients underwent 2 embolizations, 15 patients (16%) underwent 3 embolizations, and 21 patients (22%) underwent ≥4 embolizations (Tables 2 and 3). A single SRS session was performed in 64 (67%) patients, two SRS sessions in 17 patients (18%), and three to five SRS sessions in 14 (15%) patients. The median duration from last SRS session to follow-up was 32 months.

Overall procedural complications occurred in 14 (14.7%) patients. Thirteen (14%) patients experienced only minor or transient neurological complications that left no permanent morbidity. The remaining patient suffered a major neurological complication following embolization that left him severely disabled. Four (4%) patients suffered a hemorrhage during the follow-up period. One expired shortly thereafter. Thirty-eight (40%) patients had a radiographically confirmed obliteration. AVM obliteration was confirmed on DSA in 31 patients (32%) and MRA in 7 patients (8%).

**Figure 2:**
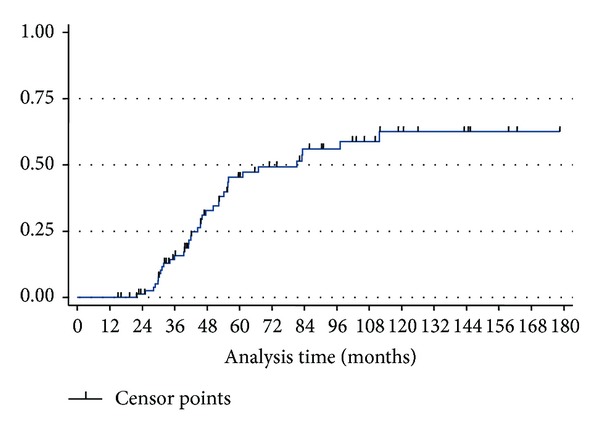
Plot showing AVM obliteration rate versus time after treatment.

Statistical analysis is detailed in Tables 4 and 5. In multivariate logistic regression analysis, larger AVM size, deep venous drainage, and increasing number of embolization or SRS sessions were negative predictors of complete obliteration. The number of embolizations correlated positively with the number of stereotactic radiosurgeries (*P* < 0.005). We analyzed patients who had a favorable outcome (complete obliteration and no neurologic deficits). Those with less than 4 embolization treatments (OR = 0.26, *P* = 0.036) and without deep venous drainage (OR = 0.22, *P* = 0.001) were significantly more likely to have favorable outcome. The postradiosurgery actuarial rate of obliteration was 36% at 3 years, 45% at 5 years, 56% at 7 years, and 63% at 10 years (Figure 2).

## 4. Discussion 

In AVMs deemed inappropriate for surgical resection, SRS is a well-established treatment alternative. Large series have reported excellent results with occlusion rates as high as 84% at 2-year angiographic follow-up [6]. Pollock et al. [7] reported a 73% rate of complete obliteration and a 14% rate of major complications. In their study, the rate of hemorrhage after treatment was 8% over a 2–5 year follow-up period. Along similar lines, Yashar et al. [8] reported a 4-year radiographic obliteration rate of 67% with a hemorrhage rate of approximately 8%. With increasing clinical experience, several predictors of outcome after SRS were identified including AVM nidus volume, geometry of nidus distribution, eloquence of surrounding brain, and the presenting symptom of hemorrhage [5].

Despite overall high obliteration rates and low complication rates, SRS efficacy remains severely limited in large and complex AVM. In fact, SRS treatment efficacy and safety profile diminish sharply with increasing AVM size [4]. In a series of 30 large AVMs, Miyawaki et al. [9] noted only a 23% obliteration rate when utilizing a LINAC dose of 16 Gy with 22% of these cases requiring surgical intervention due to symptomatic radiation necrosis. Using LINAC with a mean dose >10 Gy, Ellis et al. [10] reported an obliteration rate of 44% in AVMs larger than 10 cm^3^. Results from single-session Gamma Knife Radiosurgery (GKR) have been similarly discouraging for large AVM. Pan et al. [11] reported only a 50% obliteration rate in 76 AVMs larger than 10 cm^3^ (mean dose 17 Gy). Furthermore, the authors reported that 49% of their patients had developed moderate to severe radiation-related edema with 6.3% having treatment related symptomatic complications.

Given the poor efficacy of SRS in large and complex AVM, neurosurgeons have attempted to develop alternative treatment strategies. Our center and others have utilized endovascular embolization to reduce the volume of large AVM in an attempt to improve SRS efficacy. With a reduction in the size of these lesions by embolization, SRS may potentially prove as effective as in initially small AVM treated with appropriate SRS margin dosages. Mathis et al. [12] embolized 24 AVMs with a mean initial volume of 37 cm^3^ prior to GKR and noted a 50% obliteration rate with only 4% morbidity. In their series, procedural morbidity was exclusively related to radiosurgery. In another series, Mizoi et al. [13] treated 14 patients with AVMs >3 cm (mean volume 17.9 cm^3^) with particle embolization followed by GKR (19.2 Gy) and reported a 38% obliteration rate with an 11% permanent morbidity rate (related exclusively to the endovascular embolization). In these initial reports, particulate agents such as PVA were utilized. These agents have several drawbacks mainly related to treatment durability and technical complications. Since then, newer embolic agents with improved penetrance, selectivity, and safety profile have been developed. Gobin et al. [14] reported their experience with NBCA embolization followed by LINAC (25 Gy) in large AVM (mean initial volume 22 cm^3^). The authors were able to achieve a cure in 60% of patients with a 12.6% morbidity rate due primarily to the endovascular procedure. More recently, Blackburn et al. [15] reported an impressive 84% obliteration rate in 19 patients with AVM (average size of 20.1 cm^3^) treated with NBCA embolization (average of 2.1 sessions) followed by SRS (mean dose of 17.9 Gy at the 50% isodense line). The procedural morbidity rate was 19% in their series. Blackburn reported a 7% permanent morbidity rate per endovascular embolization and 5% permanent morbidity rate per SRS session.

Our experience with embolization prior to SRS has been positive, albeit with a more modest 40% obliteration rate. Our criteria for radiographic AVM cure included only patients in whom a frank obliteration was documented on DSA or, alternatively, on MRA in those unable or unwilling to undergo catheter angiography. We also attribute this lower angiographic cure rate to the higher proportion of patients with SM grade IV/V lesions and the larger median AVM size in our study. Median AVM volume was as high as 28 cm^3^ and nearly 50% of all lesions were SM grade IV and V.

Our protocol had a safety profile with a rate of morbidity of 14%. Most importantly, although 29% of patients presented with a ruptured AVM, only 4 (4%) patients experienced a posttreatment hemorrhage. This low rate of hemorrhage should be interpreted in light of the poor natural history of the AVMs included in our study. As such, young patients, known to have a particularly high rupture risk as demonstrated by Laakso et al. [16], accounted for up to 50% of the study population. Many patients also had larger symptomatic lesions in or abutting eloquent locations with medically intractable epilepsy or continued neurologic decline. The risk of open surgery is prohibitively high in these patients, and without preradiosurgical embolization many would not have been treatable with SRS alone due to the risks of high dose SRS radiation necrosis.

Despite the positive results previously reported by centers undertaking embolization, the use of embolization as an adjunctive tool in the treatment of large AVMs remains controversial. Some investigators reported that embolization may decrease obliteration rates and increase treatment morbidity [17]. Andrade-Souza et al. [17] retrospectively matched 47 patients who underwent Embo/SRS with 47 patients treated with SRS alone. They found an obliteration rate of 47% with Embo/SRS compared to 70% with SRS alone. However, this study is severely limited by the difficulty in matching a complex group of patients retrospectively, which may have introduced significant bias into the analysis. The authors also theorized that the lower obliteration rate observed with preradiosurgical embolization was attributable to the recanalization of the embolized portion of the nidus as well as the difficulty with radiosurgical planning since embolization may convert a fairly uniform geometric target into a poorly defined target with multiple irregular components. However, newer liquid embolic agents, such as Onyx, offer significant improvements in these areas which may theoretically increase treatment success.

Other groups have utilized different radiosurgical strategies to manage large unresectable AVM. Karlsson et al. [18] presented their data with planned staged SRS beginning with a low initial dose for volume reduction followed several years later by a follow-up SRS treatment. They treated 19 AVMs with an average volume of 16 cm^3^ and achieved an obliteration rate of 68%, with a 7% risk of morbidity and a 7% annual risk of posttreatment hemorrhage. Using the same strategy in a series of 41 patients with large AVM (average volume of 13.8 cm^3^), Foote et al. [19] achieved an obliteration rate of 59%, with a 2% permanent SRS-related morbidity and a 1.5% annual risk of posttreatment hemorrhage. Purely volume-staged SRS is a more recently reported approach with prospectively planned follow-up SRS sessions on separate portions of the AVM at six-month intervals. Richling et al. [20] reported their initial results of volume-staged SRS with 28 large AVMs (average volume of 24.9 cm^3^) achieving a 33% obliteration rate with only a 4% complication rate. A fairly similar obliteration rate (35%) was reported by the same group in a follow-up report that included 48 patients with large AVM [21]. The authors cautioned against the use of preoperative embolization, though nearly half of their patients received some form of embolization. Of their prospectively planned stereotactic staged radiosurgery, the same authors reported a 36% actuarial obliteration rate at 5 years and a 56% obliteration rate at 10 years when including salvage treatments. Importantly, 10 hemorrhages with 5 deaths occurred during follow-up, highlighting the main limitation of this treatment strategy, namely, the slow and delayed response of high risk AVMs to therapy.

We believe that the use of endovascular embolization remains a valuable option as an adjunctive modality for large AVM. We achieved a fairly reasonable obliteration rate, which could potentially improve with further angiographic followup. Furthermore, at the time of embolization, we were able to treat high-risk fistulas and proximal flow-related aneurysms as reflected in our low posttreatment hemorrhage rate. Endovascular embolization may therefore reduce the risk of hemorrhage during the treatment latency period. Additionally, our treatment success came with a low complication rate that compares favorably to previous studies. The question of whether this complication rate is better than the natural history of these lesions left untreated cannot be definitively answered in the present study. For some patients included in this study, SRS without prior embolization or even surgical resection could have been potential alternatives. Future advances in endovascular techniques and liquid embolic agents will undoubtedly improve the safety and efficacy of AVM embolization. Accordingly, recent data may suggest that Onyx allows more permanent lesion obliteration, with better visualization on SRS planning, MR imaging, and less embolic complications; however, this remains controversial [22].

## 5. Conclusions

SRS preceded by adjuvant embolization is a controversial yet potentially efficacious treatment strategy in patients with large AVMs that are not amenable to surgical excision. We were able to achieve complete AVM obliteration in a significant number of patients. Larger prospective studies are needed to explore the long-term safety and efficacy of this approach.

## Figures and Tables

**Table 1:** 

	*N*	%
Gender		
M	41	42%
F	54	58%

Age		
Median in yrs	38.54	
0–21	18	18.90%
22–40 yrs	32	33.70%
40–50 yrs	22	23.20%
50+	23	24.20%

AVM side		
L	54	57%
R	41	43%

Initial hemorrhage		
N	68	71%
Y	27	29%

Initial seizure		
N	58	61.10%
Y	37	38.90%

Initial neurological exam		
Intact	74	78.00%
Mild/moderate deficit	18	18.90%
Severe deficit (i.e., unresponsive)	3	3.10%

Location	*N*	%

Frontal	19	20.00%
Temporal	24	25.30%
Parietal	26	27.40%
Occipital	9	9.40%
Cerebellum	8	8.40%
Thalamus, brainstem	9	9.40%

Size		
Medium (3–6 cm)	80	84.20%
Large (>6 cm)	15	15.80%

Eloquence		
Noneloquent tissue	24	25.20%
Eloquent tissue	71	74.80%

Venous drainage		
Superficial	44	46.30%
Deep	51	53.70%

SM grade		
3	48	50.50%
4	38	40.00%
5	9	9.50%

Pollock-Flickinger score		
0–2.25	23	24.2%
2.25–3.5	24	25.3%
3.5–4.75	24	25.3%
>4.75	24	25.3%

Radiosurgery AVM scale		
1	12	12.6%
2	46	46.3%
3	28	29.4%
4	11	11.6%

**Table 2:** 

Total number of embolizations	*N* (mean 2.44)	%
1	34	35.80%
2	25	26.30%
3	15	15.80%
4+	21	22.10%

Time to complete multiple embolizations	Avg months (mean 7.7)	*N*

2 embolizations	1.9	28
3 embolizations	7.8	17
4 embolizations	11.3	15
5+ embolizations	30.8	11

Type of embolization	*N*	%

NBCA	230	83%
Onyx	46	17%

Total number of radiation treatments	*N*	%

1	64	67.30%
2	17	17.90%
3+	14	14.70%

**Table 3:** 

SRS Tx	Total embo Tx						
	1	2	3	4	5	6	7
1	28	17	10	7	1	1	0
2	5	4	2	5	1	0	0
3	1	3	2	0	2	0	1
4	0	1	0	1	0	1	1
5	0	0	1	0	0	0	0

Spearman's rho 0.403, *P* < 5.982*e* − 6.

**Table 4:** 

	Total (%)	No. w/residual AVM (%)		No. w/100% obliteration (%)		*P* value
Sex, *n* (%)						0.396
M	41	21	51.20%	20	48.80%	
F	54	36	66.70%	18	33.30%	

Age, *n* (%)						0.795
0–21	18	10	55.60%	8	44.40%	
22–40 yrs	32	21	65.60%	11	34.40%	
40–50 yrs	22	14	63.60%	8	36.40%	
50+	23	12	52.20%	11	47.80%	

Location						0.252
Frontal	19	14	73.70%	5	26.30%	
Temporal	24	13	54.20%	11	45.80%	
Parietal	26	12	46.20%	14	53.80%	
Occipital	9	7	87.80%	2	22.20%	
Cerebellum	8	4	50%	4	50%	
Thalamus, brainstem	9	7	87.80%	2	22.20%	

Size, *n* (%)						0.022
Medium (3–6 cm)	80	44	55%	36	45%	
Large (>6 cm)	15	13	86.70%	2	13.30%	

Venous drainage, *n* (%)						<0.001
Superficial	44	18	40.90%	26	59.10%	
Deep	51	39	76.50%	12	23.50%	

Smoker? *n* (%)						0.664
Y	35	20	57.10%	15	42.90%	
N	60	37	61.70%	23	38.30%	

Hypertension? *n* (%)						0.669
Y	18	11	61.10%	7	38.90%	
N	77	46	59.70%	31	40.30%	

**Table 5:** 

	Total (*n*)	No w/residual AVM		No. w/100% obliteration		*P* value
		%		%		
Initial hemorrhage						0.926
Y	27	16	59%	11	41%	
N	68	40	59%	28	41%	

Initial seizure						0.731
Y	37	25	68%	12	32%	
N	58	31	53%	27	47%	

Initial neurological exam						0.25
Intact	74	45	61%	29	39%	
Mild/moderate deficit	18	8	44%	10	56%	
Severe deficit	3	3	100%	0	0%	

Total number of embolizations						0.017
1	34	18	53%	16	47%	
2	25	13	52%	12	48%	
3	15	8	53%	7	47%	
4+	21	17	81%	4	19%	

Total number of radiation treatments						0.025
1	64	33	52%	31	48%	
2	17	12	72%	5	29%	
3+	14	12	79%	2	14%	

## Abbreviations

AVM: Arteriovenous malformations
SM: Spetzler-Martin
SRS: Stereotactic radiosurgery
GKR: Gamma Knife Radiosurgery.
